# Glial Cell Dynamics in Neuroinflammation: Mechanisms, Interactions, and Therapeutic Implications

**DOI:** 10.3390/biomedicines14010115

**Published:** 2026-01-06

**Authors:** Mario García-Domínguez

**Affiliations:** 1 Program of Immunology and Immunotherapy, CIMA-Universidad de Navarra, 31008 Pamplona, Spain; mgdom@unav.es; 2 Department of Immunology and Immunotherapy, Clínica Universidad de Navarra, 31008 Pamplona, Spain; 3 Centro de Investigación Biomédica en Red de Cáncer (CIBERONC), 28029 Madrid, Spain

**Keywords:** neuroinflammation, glial cells, cytokines, demyelination, blood–brain barrier, pathological stimuli

## Abstract

Neuroinflammation is a defining feature of many neurological disorders, including neurodegenerative diseases, traumatic brain injury, and demyelinating conditions. Glial cells play a central role in this process by initiating, modulating, and resolving inflammatory responses in the CNS. This review examines the diverse roles of glial cells in neuroinflammation, focusing on their molecular and cellular interactions, context-dependent activation states, and phenotypic plasticity. It discusses how microglial activation can result in both neuroprotective and neurotoxic effects, while astrocytes contribute to immune regulation, blood–brain barrier integrity, and neuronal survival. The review also highlights interactions between glial cells and peripheral immune components, which may exert synergistic or antagonistic effects. Finally, it outlines emerging preclinical and clinical strategies targeting glial pathways to modulate several neuroinflammatory outcomes, emphasizing that a detailed understanding of glial dynamics is essential for developing effective CNS therapies.

## 1. Introduction

Neuroinflammation is a complex process that is involved in numerous diseases of the nervous system [1,2], which include acute injuries such as traumatic brain injury (TBI) [3] and stroke [4], as well as chronic neurodegenerative disorders like Alzheimer’s disease (AD) [5], Parkinson’s disease (PD) [6], multiple sclerosis (MS) [7], and amyotrophic lateral sclerosis (ALS) [8]. In contrast to peripheral inflammation, neuroinflammation is coordinated in the microenvironment of the central nervous system (CNS), in which specialized immune-competent cells, known as glial cells, play a pivotal regulatory role [9]. Glial cells which include microglial cells, astrocytes, oligodendrocytes, and their progenitors, are not only the primary mediators of the CNS innate immune response but also the main contributors to the homeostasis, synaptic remodeling, and neuronal support under physiological conditions [10,11].

Upon exposure to pathological stimuli or injury, glial cells undergo phenotypic and functional modifications, a phenomenon broadly referred to as glial activation [12]. This dynamic response encompasses coordinated changes in morphology, transcriptional and translational circuits, secretory functions, and intercellular communication with neurons as well as other glial cell types [13]. Glial activation serves as a defining characteristic of the CNS’s response to a diverse array of insults, such as trauma, infection, ischemia, and neurodegenerative processes [14].

The first line of defense against damage or pathogen invasion is provided by microglial cells, the CNS’s resident innate immune cells and main phagocytes [15]. Due to their high degree of plasticity, microglial cells can quickly switch between two activation states. Traditionally, these have ranged from pro-inflammatory phenotype (M1-like) that can exacerbate neuronal damage to anti-inflammatory (M2-like) that aid in tissue regeneration and repair [16]. Numerous environmental cues, including cytokines, pathogen-associated molecular patterns, and damage-associated molecular patterns (DAMPs), influence this spectrum of activation [17].

The most predominant glial cell type, astrocytes, are also fundamental for maintaining homeostasis in the CNS and responding to injury [18]. When astrocytes are activated, they exhibit reactive astrogliosis, which is typified by changes in gene expression profiles, hypertrophy, and upregulation of intermediate filaments like glial fibrillary acidic protein (GFAP) [19]. Reactive astrocytes have many functions, including regulating extracellular ion and neurotransmitter concentrations, secreting numerous pro- and anti-inflammatory mediators, influencing synaptic plasticity, and influencing neuronal metabolism and permeability of the blood–brain barrier (BBB) [20]. Beyond their crucial role in mediating immune signaling within the CNS, oligodendrocytes are commonly known for their function in the development and upkeep of myelin sheaths surrounding axons [21]. By releasing a variety of cytokines, oligodendrocytes can contribute to neuroinflammation under pathological conditions. They might also participate in remyelination and axonal support processes [22]. Furthermore, compared to earlier estimates, their interactions with other glial cells point to a more integrated role in the larger glial response to CNS insult [23].

A dynamic and complex network of interactions, essential for the initiation and resolution of CNS immune responses, is exemplified by the interplay among glial subtypes during neuroinflammation [24]. The release and reception of cytokines, extracellular vesicles (such as exosomes and microvesicles), and direct physical interactions through specific cell–cell contact sites are all part of this bidirectional communication, which involves a complex interplay of signaling pathways [25]. In this neuroimmune network, each type of glial cell shapes both local and systemic responses to injury or disease through unique but interdependent roles [26].

In order to describe the role of glial cells in the development, progression, and resolution of neuropathological conditions, the idea of disease-associated glial cells has gained popularity [27]. Molecularly distinct glial subpopulations that arise in response to disease-specific perturbations, such as demyelination in MD or protein aggregation in other neurodegenerative disorders, have been identified thanks to high-definition techniques like scRNA-seq (single-cell RNA sequencing) and spatial transcriptomics [28,29]. These transcriptionally altered states are characterized by changes in immune signaling, metabolic pathways, synaptic modulation, and phagocytic activity, and they represent context-dependent adaptations rather than just reactive phenotypes [30]. Specifically, single-cell RNA sequencing (scRNA-seq) studies have revealed that the classical M1/M2 framework is overly simplistic and fails to accurately represent the full transcriptional and functional diversity of microglial cells. Rather than existing as binary activation states, microglia display a dynamic continuum of context-dependent phenotypes that are highly sensitive to developmental stage, aging, regional CNS environment, and pathological insults [31,32]. These include homeostatic microglia that maintain synaptic pruning and tissue surveillance, developmental- and aging-associated subpopulations with stage-specific transcriptional programs, disease-associated microglia (DAM) that emerge in neurodegenerative conditions, and injury-induced phenotypes that respond to tissue damage. Beyond these broad categories, scRNA-seq analyses have further identified microglial subsets with specialized functions, including antigen-presenting cells capable of modulating adaptive immune responses, interferon-responsive populations that participate in antiviral defense, metabolically distinct microglia optimized for energy-intensive processes, and highly proliferative subsets involved in local tissue repopulation. Importantly, these subpopulations exhibit regional specialization within the CNS, reflecting the unique microenvironmental cues of different brain and spinal cord regions [31,32].

Analogously, astrocytes also exhibit remarkable heterogeneity in response to pathological stimuli. Under disease, scRNA-seq studies have identified multiple astrocytic subtypes defined by distinct metabolic pathways, differential expression of neurotrophic and synaptogenic factors, and specialized roles in modulating neuronal activity, synaptic remodeling, and blood–brain barrier integrity [33]. Certain astrocyte populations contribute to protective processes, such as supporting neuronal survival and facilitating synaptic recovery, whereas others may adopt maladaptive profiles that exacerbate inflammation or synaptic dysfunction [33].

Glial cell heterogeneity, plasticity, and context-dependent functions are now understood thanks to many developments in molecular and imaging technologies, which have also revealed intricate regulatory networks that control how these cells behave in health and illness. As a result, glial cell targeting offers an effective therapeutic approach to reduce neuroinflammatory responses and encourage neuroprotection and repair [34]. Current research focusses on pharmacological approaches, such as gene-targeting strategies, biologics, and small-molecule modulators, to modify glial activation, reduce neuroinflammation, and support neuroprotection and repair [35,36,37,38,39].

This review provides a focused analysis of recent discoveries on the temporal and spatial dynamics of glial cell activation and their interactions during neuroinflammation. Centered on the question of how these dynamics shape CNS inflammatory responses and impact disease progression, it emphasizes mechanistic pathways through which glial cells detect, propagate, and resolve neuroinflammatory signals, highlighting those most relevant for translational and therapeutic applications. Rather than attempting a comprehensive catalog of all facets of glial biology, this review emphasizes mechanisms with demonstrated functional significance and translational relevance, while more speculative or less-established pathways are noted as directions for future research. By integrating evidence from cellular, molecular, and translational studies, this review delineates the relative significance of individual mechanisms, establishes a coherent framework for understanding glial contributions to CNS inflammation, and highlights their potential as targets for novel interventions aimed at attenuating neuroinflammation and improving clinical outcomes.

## 2. Glial Cell Types and Their Functions

Maintaining neural homeostasis, promoting neuronal function, and regulating synaptic activity all depend on glial cells, a varied subset of non-neuronal cells found in the CNS. Microglial cells, astrocytes, and oligodendrocytes are examples of glial cells, and each has distinct baseline functions that are essential for maintaining the integrity of the CNS [10]. An outline of their basic roles will be given in this section.

### 2.1. Microglial Cells

In contrast to other glial and peripheral immune cells, microglial cells are the resident innate immune cells of the CNS and are derived from primitive myeloid progenitors that invade the brain parenchyma during embryogenesis [40,41]. Microglial cells exhibit a dynamic, ramified morphology under physiological conditions. Their fine processes continuously extend and retract to actively survey the surrounding neural microenvironment. They can identify alterations in tissue homeostasis, such as indications of cellular stress, injury, infection, or changed synaptic activity, due to their motility [42]. Microglial cells can quickly shift into an activated state in response to these cues, which is characterized by morphological changes (e.g., retraction of processes and enlargement of the cell body), upregulation of surface antigens such as MHC-I (major histocompatibility complex class I) and MHC-II (major histocompatibility complex class II), and the production of reactive oxygen species (ROS), nitric oxide (NO), and some pro-inflammatory cytokines and chemokines (e.g., IL-1β, TNF-α, CCL2, and CXCL10) [43,44].

Activated microglial cells play a central role in phagocytosing apoptotic cells, cellular debris, and invading pathogens, thereby maintaining CNS integrity [45]. Crucially, they also have an impact on neurodevelopment by means of synaptic pruning, which is the process of removing weak or superfluous synaptic connections, especially during essential stages of postnatal brain maturation [46]. Through ligand-receptor pairs like CX3CL1-CX3CR1 and CD200-CD200R, microglial cells engage with neurons and other glial cells, assisting in their homeostatic, non-inflammatory state [47,48]. Many neuropathological disorders, including neurodegenerative diseases including AD, PD, and MS, are increasingly linked to dysregulation of microglial function. In these conditions, microglial cells develop persistently pro-inflammatory phenotypes that worsen neuronal damage [49].

### 2.2. Astrocytes

The most abundant glial cell type in the CNS, astrocytes are key specialized cells that carry out a variety of homeostatic, metabolic, and regulatory tasks necessary for healthy CNS development and operation [50]. Astrocytes exhibit a characteristic star-shaped morphology and are distinguished by the expression of GFAP. They are ideally positioned anatomically to regulate neurovascular and synaptic activity because they extend a variety of fine processes that envelop synapses, blood vessels, and other neural elements [51]. The maintenance of the BBB integrity, where perivascular astrocytic end-feet interact with pericytes and endothelial cells to control permeability and shield the CNS from circulating pathogens and toxins, is one of their most important functions [52]. In order to maintain appropriate neuronal excitability and avoid excitotoxicity, astrocytes are also essential for controlling extracellular ion concentrations, mainly K^+^, which they buffer via inward-rectifying K^+^ channels [53]. By absorbing excess neurotransmitters such as glutamate and GABA (γ-aminobutiric acid) through high-affinity transporters (such as GLT-1 -glutamate transporter 1- and GLAST -glutamate aspartate transporter-), astrocytes participate in the tripartite synapse in the synaptic domain. They then transform glutamate into glutamine, which is then recycled back to neurons [54]. Additionally, astrocytes modulate synaptic plasticity, long-term potentiation (LTP), and learning and memory processes by releasing gliotransmitters such as ATP (adenosine triphosphate), D-serine, and glutamate in a Ca^2+^-dependent manner [55].

Additionally, they are essential for energy metabolism because they use aerobic glycolysis to convert glucose to lactate, which is then sent to neurons via the astrocyte-neuron lactate shuttle (ANLS) [56]. Moreover, it has been reported that astroglial activation and ROS production occur during neuroinflammatory processes [57]. Astrocytes support the definition of cortical and subcortical boundaries, promote axon growth, and direct neuronal migration during development [58]. In reaction to CNS injury or disease, astrocytes undergo a process called reactive astrogliosis, which includes transcriptional and morphological alterations like hypertrophy, GFAP upregulation, and the release of inflammatory mediators [59]. Chronic astrogliosis might also prevent axonal regeneration and lead to pathological remodeling of neural circuits, even though reactive astrocytes can perform many neuroprotective tasks by creating glial scars and preventing damage from spreading [60].

### 2.3. Oligodendrocytes

To facilitate rapid saltatory conduction of action potentials and ensure effective neural communication over long distances, oligodendrocytes are responsible in the formation and maintenance of myelin sheaths around axons in the CNS [61]. Oligodendrocytes are derived from oligodendrocyte precursor cells (OPCs), which undergo tightly controlled processes of proliferation, migration, and differentiation to reach their mature and myelinating state. OPCs originate in the ventricular zones during embryonic development and continue into adulthood [62]. Mature oligodendrocytes produce myelin sheaths made of densely packed, multilamellar, lipid-enriched membranes by extending certain processes that enwrap discrete axonal segments. This structure promotes long-term axonal integrity and viability by facilitating saltatory propagation between nodes of Ranvier and increasing the conduction velocity of nerve impulses. It also provides trophic and metabolic support to the ensheathed axon [63].

**Figure 1:**
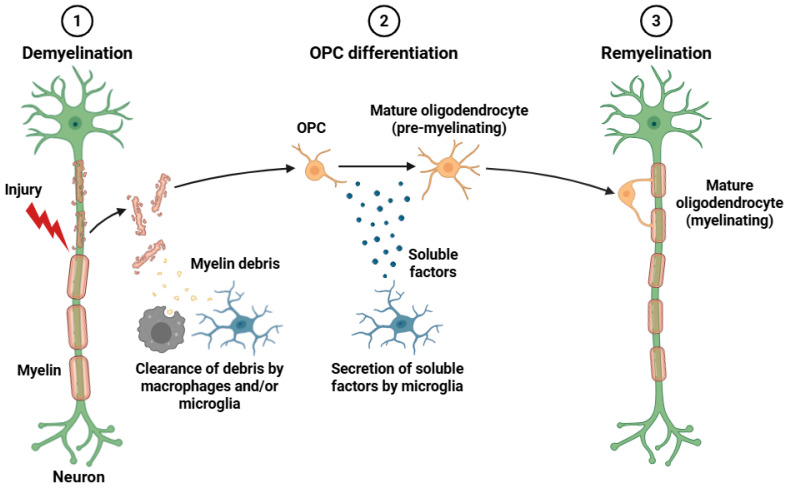
This image illustrates the process of remyelination in the CNS through three sequential stages. The loss of myelin sheaths caused by neuronal injury during demyelination first accumulates myelin debris, which is then removed by macrophages and/or microglial cells. Microglial cells secrete several soluble factors that promote OPCs differentiation into mature pre-myelinating oligodendrocytes in the second stage. These differentiated oligodendrocytes ultimately regenerate the myelin sheaths surrounding axons, thus reestablishing proper neuronal function during the remyelination phase. In demyelinating disorders such as MS, this process is critical for the restoration of neural integrity and functional recovery. Abbreviations: OPC (oligodendrocyte precursor cell).

Oligodendrocytes react to neuronal activity by adjusting the amount and thickness of myelination, which affects neural plasticity and circuit refinement. Myelination is activity-dependent [64]. In addition to their ability to myelinate, oligodendrocytes express a variety of adhesion molecules, neurotransmitter receptors (e.g., NMDA -N-methyl-D-aspartate and AMPA -α-amino-3-hydroxy-5-methyl-4-isoxazolepropionic acid-), and ion channels that facilitate two-way communication with astrocytes and neurons. Demyelinating disorders, like MS, are characterized by disruption of oligodendrocyte function or myelin integrity. In these disorders, immune-mediated attacks cause myelin to be lost, which results in impaired conduction, neurodegeneration, and severe neurological deficits [65]. Chronic MS and other myelin-related disorders are characterized by incomplete or dysfunctional remyelination (Figure 1), the process of regenerating new myelin sheaths from surviving OPCs [66]. Furthermore, there is growing evidence that oligodendrocyte morphology is linked to neurodevelopmental and psychiatric disorders with altered myelination patterns and white matter abnormalities, like major depressive disorder (MDD), autism spectrum disorder (ASD), and schizophrenia [67,68].

## 3. Mechanisms of Glial Activation in Neuroinflammation

In the CNS, glial cells are positioned as sentinels and effectors of neuroinflammatory responses through the complex and dynamic process of glial activation, which incorporates a broad range of molecular mechanisms [10,11,69]. Innate immune receptors, transcriptional reprogramming, metabolic changes, intercellular communication, and interactions with peripheral immune cells are all part of the signaling networks that control glial cells during neuroinflammation, all of which influence the progression of CNS pathologies [17,19,21].

### 3.1. Role of Microglial Cells in Neuroinflammation

**Figure 2:**
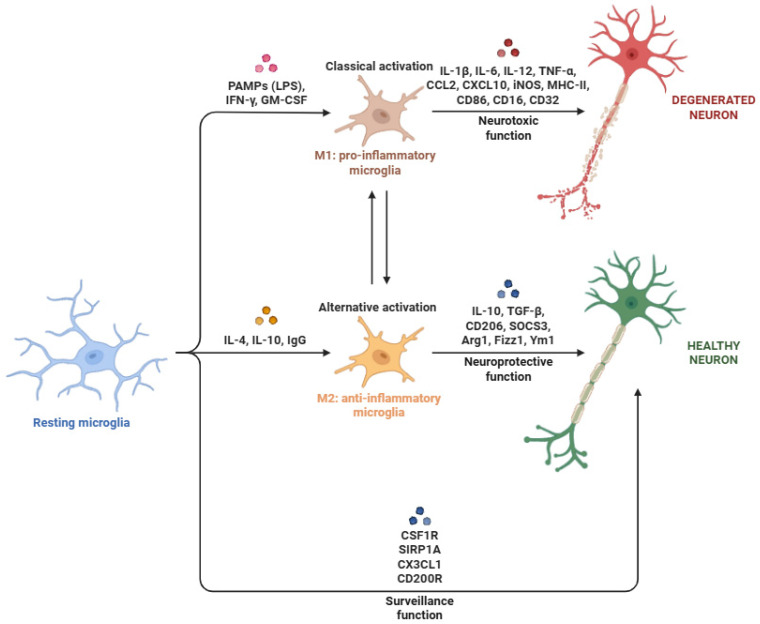
Microglial polarization and its impact on neuronal outcomes. Resting microglial cells maintains homeostasis through surveillance functions mediated by molecules like CSF1R, SIRP1A, CX3CL1, and CD200R. Upon activation, microglial cells can polarize into either a pro-inflammatory (M1) or anti-inflammatory (M2) phenotype. M1 microglial cells are induced by classical activation signals such as PAMPs (e.g., LPS), IFN-γ, and GM-CSF, secrete several pro-inflammatory mediators including IL-1β, IL-6, IL-12, TNF-α, CCL2, CXCL10, and iNOS, and express MHC-II, CD86, and CD16/32, leading to neurotoxic effects and neuronal degeneration. In contrast, alternative activation by IL-4, IL-10, or IgG promotes an M2 phenotype characterized by anti-inflammatory and neuroprotective responses, including the production of IL-10, TGF-β, and expression of CD206, SOCS3, Arg1, Fizz1, and Ym1, supporting neuronal survival and maintaining healthy neurons. The balance between M1 and M2 phenotypes determines the outcome of neuroinflammation and is critical in neurodegenerative and demyelinating diseases. Abbreviations: PAMP (pathogen-associated molecular pattern), LPS (lipopolysaccharide), IFN-γ (interferon gamma), GM-CSF (granulocyte-macrophage colony-stimulating factor), IL-4 (interleukin 4), IL-10 (interleukin 10), IgG (immunoglobulin G), IL-1β (interleukin 1 beta), IL-6 (interleukin 6), IL-12 (interleukin 12), TNF-α (tumor necrosis factor alpha), CCL2 (C-C motif chemokine ligand 2), CXCL10 (C-X-C motif chemokine ligand 10), iNOS (inducible nitric oxide synthase), MHC-II (major histocompatibility complex class II), CD86 (cluster of differentiation 86), CD16 (cluster of differentiation 16), CD32 (cluster of differentiation 32), IL-10 (interleukin-10), TGF-β (transforming growth factor beta), CD206 (cluster of differentiation 206), SOCS3 (suppressor of cytokine signaling 3), Arg1 (arginase 1), Fizz1 (found in inflammatory zone 1), Ym1 (chitinase-like protein 3), CSF1R (colony-stimulating factor 1 receptor), SIRP1A (signal regulatory protein alpha), CX3CL1 (C-X3-C motif chemokine ligand 1), and CD200R (CD200 receptor).

Microglial cells (Figure 2) are uniquely adapted to sense and respond to perturbations in CNS homeostasis. Their immunosurveillance capacity relies on many germline-encoded pattern recognition receptors (PRRs), which detect some PAMPs pathogen-associated molecular pattern) and DAMPs [70,71]. TLR2 recognizes several bacterial lipoproteins and peptidoglycans [72]; TLR4 is sensitive to lipopolysaccharide (LPS) as well as endogenous ligands like heat shock proteins and high-mobility group box 1 (HMGB1) [73]; and TLR9 detects unmethylated CpG DNA (deoxyribonucleic acid) motifs [74]. TLRs (Toll-like receptors) use their Toll/interleukin-1 receptor (TIR) domains to dimerize and identify adaptor proteins after ligand engagement. With the exception of TLR3, which signals through TRIF (TIR-domain-containing adapter-inducing interferon-β), most TLRs employ MyD88 (myeloid differentiation primary response 88) as their primary adaptor. TLR4 uses both adaptors, depending on the subcellular signaling compartment [75].

MyD88 engagement promotes the recruitment of TNF receptor-associated factor 6 (TRAF6) and IL-1 receptor-associated kinases (IRAKs), promoting the activation of TAK1 (TGF-β-activated kinase 1) [76]. TAK1 subsequently phosphorylates the IκB kinase (IKK) complex (IKKα, IKKβ, and IKKγ), leading to IκBα phosphorylation and its ubiquitination and proteasomal degradation. This enables nuclear translocation of NF-κB (nuclear factor kappa-light-chain-enhancer of activated B cells), typically the p65/p50 heterodimer, and transcription of several pro-inflammatory genes [77], including chemokines (CCL2, CCL5, and CXCL10), interleukins (IL-1β, IL-6, and IL-12), and TNF-α that coordinate immune cell recruitment and activation [78]. In parallel, TRIF-dependent signaling activates interferon regulatory factors (IRF3/7), inducing type I interferons (IFN-α/β) and JAK (Janus kinase)-STAT (signal transducer and activator of transcription) signaling, thus amplifying inflammation [79]. Activated microglia also upregulate MHC-I and MHC-II molecules and costimulatory proteins (CD80 and CD86), facilitating antigen-presenting cell (APC) functions, particularly under chronic neuroinflammatory conditions [80].

The NLRP3 (NOD-like receptor family pyrin domain-containing 3) inflammasome is a key downstream effector of microglial activation. Its activation requires a priming signal through TLR-NF-κB-dependent transcription of pro-IL-1β, pro-IL-18, and inflammasome components, followed by an activation signal driven by cellular stressors such as K^+^ efflux, ROS accumulation, mitochondrial dysfunction, or lysosomal rupture [81]. These signals induce NLRP3 oligomerization and recruitment of pro-caspase-1 and ASC (apoptosis-associated speck-like protein containing a CARD) [82]. Caspase-1 activation results in maturation and release of IL-1β and IL-18 via gasdermin D-mediated pore formation, leading to pyroptosis, a pro-inflammatory form of cell death that sustains CNS immune activation [83,84].

Extracellular ATP is a potent activator of microglial purinergic receptors, particularly the ionotropic P2X7 receptor [85]. P2X7 activation induces Ca^2+^ influx and K^+^ efflux, the latter being a critical upstream signal for NLRP3 inflammasome activation [86]. Continued P2X7 signaling promote pannexin-1-dependent pore formation, reinforcing autocrine and paracrine inflammatory loops [87]. Metabotropic purinergic receptors such as P2Y6 (UDP-responsive) and P2Y12 (ADP-responsive) regulate microglial chemotaxis and phagocytosis via PI3K/AKT/Rac1-mediated cytoskeletal remodeling, supporting targeted migration and debris clearance [88,89].

Neuron-glia crosstalk fine-tunes microglial activation. The CX3CL1-CX3CR1 axis, involving neuronal fractalkine and its microglial receptor, is essential for synaptic pruning and microglial homeostasis [90]. Disruption of this signaling mechanism promotes a pro-inflammatory microglial phenotype characterized by increased TNF-α, IL-1β, and inducible nitric oxide synthase (iNOS), thereby compromising neuroprotection [91]. Microglial activation is accompanied by metabolic reprogramming from mitochondrial oxidative phosphorylation (OXPHOS) to aerobic glycolysis [92], driven by stabilization of hypoxia-inducible factor 1-alpha (HIF-1α), which induces glycolytic enzymes and pro-inflammatory genes [93,94]. Alternatively, the activation of mTORC1 (mechanistic target of rapamycin complex 1) further supports inflammation by suppressing autophagy and enhancing biosynthetic pathways [95]. Increased flux through the pentose phosphate pathway generates NADPH, fueling NOX2 (NADPH oxidase 2)-dependent ROS production [96]. ROS regulate inflammasome activation, redox-sensitive transcription factors (NF-κB and AP-1), and oxidative damage [97]. In parallel, NF-κB- and STAT1-dependent upregulation of iNOS increases nitric oxide (NO) production, leading to peroxynitrite formation and disruption of mitochondrial, cytoskeletal, and neuronal integrity [98].

Finally, microglial expression of MHC-I, MHC-II, and costimulatory ligands such as CD40 enables interactions with CD8^+^ and CD4^+^ T cells [99]. IFN-γ released by activated T cells potently enhances microglial activation, promoting the release of neurotoxic mediators such as TNF-α, NO, and glutamate, while reinforcing MHC-II expression [100]. Sustained microglia–T cell interactions support immunological synapse formation, continuous antigen presentation, and neuroinflammation in disorders such as MS and AD [101].

### 3.2. Role of Astrocytes in Neuroinflammation

Regarded as support glial cells in the CNS, astrocytes are now recognized as active participants in neuroinflammation, responding to direct sensing of DAMPs and PAMPs as well as to pro-inflammatory cytokines released primarily by activated microglial cells [102,103]. Astrocytes express PRRs like TLR2, TLR3, TLR4, and RAGE (receptor for advanced glycation end products), which, upon binding ligands like HMGB1, amyloid-β, or bacterial components, trigger intracellular cascades activating transcription factors essential for astrocyte reactivity, notably the JAK-STAT and NF-κB pathways (mainly STAT3) [104,105,106].

During neuroinflammation, microglia release several cytokines (such as TNF-α and IL-1β), which activate astrocytic kinases including JAKs. Activated JAKs phosphorylate STAT3 (signal transducer and activator of transcription 3), promoting its dimerization, nuclear translocation, and binding to target gene promoters, triggering a reactive astrocyte transcriptional program that upregulates cytoskeletal proteins (e.g., GFAP, nestin, and vimentin) and induces morphological changes characteristic of astrogliosis [107,108,109]. STAT3 also enhances expression of some chemokines (CCL2, CXCL1, and CXCL10), promoting leukocyte recruitment across the BBB. Concurrently, astrocyte-derived factors regulate BBB permeability and neurovascular unit integrity, involving PKC and MAPKs to disrupt tight junction proteins (claudins and occludins), while VEGF (vascular endothelial growth factor), PGE_2_ (prostaglandin E2), and MMP-9 (matrix metalloproteinase 9) are upregulated, facilitating peripheral immune cell infiltration and amplifying neuroinflammatory cascades [110,111,112].

Astrocytes are crucial for glutamate homeostasis through EAAT1 (excitatory amino acid transporter 1) and EAAT2 (excitatory amino acid transporter 2) transporters, but inflammatory conditions reduce their expression and function via transcriptional repression and post-translational modifications (e.g., nitrosylation and ubiquitination), causing extracellular glutamate accumulation, excitotoxicity, Ca^2+^ overload, mitochondrial dysfunction, protease activation, and ROS production [113,114,115]. They also secrete damage-associated molecules such as S100B and lipocalin-2 (LCN2). S100B (S100 calcium-binding protein B) exerts trophic effects at low concentrations but acts as a DAMP at high levels via RAGE, activating NF-κB signaling. LCN2 binds to and activates 24p3R, disrupting iron homeostasis, inducing pro-inflammatory genes, and promoting cytoskeletal remodeling, reinforcing reactive glial phenotypes and neurotoxicity [116,117,118,119,120].

The phenotypic heterogeneity of astrocytes has traditionally been categorized into A1 and A2 subtypes [121]. However, Escartin et al. (2021) emphasize that astrocyte reactivity should be defined via multidimensional molecular and functional profiling, underscoring that reactive states can involve both the loss of homeostatic functions and the acquisition of novel protective or deleterious properties [122]. For the purposes of simplicity, the traditional A1/A2 classification will be used here. A1 astrocytes, induced by microglia-derived IL-1α, TNF-α, and C1q, upregulate complement components such as C3, tagging synapses for elimination by microglial CR3-mediated phagocytosis. This aberrant synaptic pruning contributes to synapse loss in neurodegenerative diseases including AD, MS, and Huntington’s disease (HD) [123,124,125,126].

Astrocyte-microglia crosstalk sustains neuroinflammation through astrocyte secretion of IL-6 and GM-CSF (granulocyte-macrophage colony-stimulating factor), which activate microglia and potentiate pro-inflammatory phenotypes, establishing a feed-forward loop [127]. This activation also upregulates adhesion molecules (ICAM-1, VCAM-1, and E-selectin) on endothelial cells, facilitating immune cell adhesion and transmigration into the CNS [128]. Astrocytes themselves can express several adhesion molecules (e.g., ICAM-1), likely modulating perivascular immune interactions, while the brain endothelium is the primary site for functional ICAM-1 upregulation [129,130].

Finally, reactive astrocytes contribute to dysregulation of CNS metabolism and redox homeostasis. In neuroinflammatory conditions, astrocytes upregulate glycolytic enzymes and attenuate mitochondrial respiration, mirroring microglial metabolic reprogramming, which leads to increased lactate production that modulates neuronal excitability and survival [131]. Astrocytes also show increased expression of NADPH oxidase isoforms, contributing to ROS production, alongside dysregulated glutathione metabolism, which compromises antioxidant defenses. This redox imbalance exacerbates cellular stress and amplifies neuroinflammation [132].

### 3.3. Role of Oligodendrcytes in Neuroinflammation

In contrast to microglial cells and astrocytes, which have broad classical immune functions, oligodendrocytes (primarily responsible for producing and maintaining myelin sheaths around CNS axons) [61] also actively participate in neuroinflammatory processes [22]. Their susceptibility to inflammatory mediators, oxidative stress, and metabolic disturbances contributes to oligodendrocyte dysfunction, demyelination, and propagation of inflammatory signaling in the CNS [133]. Oligodendrocyte precursor cells (OPCs), which support oligodendrocyte replacement and remyelination, express several PRRs (including TLR2 and TLR4) [134]. Binding of DAMPs or PAMPs activates intracellular cascades such as PI3K/AKT and MAPKs (ERK1/2 -extracellular signal-regulated kinases 1 and 2-, p38, and JNK -c-Jun N-terminal kinase-) [102], downregulating myelin-related genes (MBP—myelin basic protein, PLP—proteolipid protein, and MAG—myelin-associated glycoprotein) and impairing OPC maturation [135]. Transcription factors such as c-Jun and NFAT (nuclear factor of activated T-cells) further suppress differentiation, causing remyelination failure in chronic neuroinflammation [136].

Some pro-inflammatory cytokines (e.g., IFN-γ and TNF-α) activate oligodendrocytes via JAK/STAT1 and NF-κB pathways [137,138]. IFN-γ activates JAK1/JAK2 and STAT1, inducing antigen presentation genes (e.g., MHC-I) and increasing vulnerability to cytotoxic CD8+ T cells [139,140]. TNF-α activates TNFR1 (TNF receptor 1) and canonical NF-κB signaling, upregulating pro-apoptotic molecules (TRAIL—tumor necrosis factor-related apoptosis-inducing ligand and FasL—Fas ligand) and triggering caspase-mediated apoptosis [141,142].

Oligodendrocytes are susceptible to mitochondrial dysfunction during neuroinflammation due to their high metabolic demands, which are required to maintain lipid biosynthesis for myelin production. Upon activation, microglial cells and astrocytes produce and secrete reactive nitrogen species (RNS) and ROS, which induce mitochondrial membrane depolarization, promoting mitochondrial membrane permeabilization and cytochrome c release [43,143,144]. Through the formation of the apoptosome complex, which recruits and activates initiator caspase-9, this event triggers the intrinsic apoptotic pathway, which in turn triggers the activation of effector caspases-3 and -7, which cause cell death [145]. iNOS-derived NO exacerbates oligodendrocyte apoptosis and mitochondrial dysfunction [146].

On the other hand, oligodendrocytes are highly susceptible to endoplasmic reticulum (ER) stress under conditions of chronic neuroinflammation, which leads to the activation of the unfolded protein response (UPR) through the three signaling branches: PERK (protein kinase RNA-like ER kinase), IRE1α (inositol-requiring enzyme 1 alpha), and ATF6 (activating transcription factor 6) [147]. The activation of PERK induces phosphorylation of eukaryotic initiation factor 2 alpha (eIF2α), leading to an attenuation of protein synthesis while enhancing the translation of stress-responsive transcripts, such as ATF4 (activating transcription factor 4), and DDIT3 (DNA damage-inducible transcript 3), which mediate pro-apoptotic signaling pathways [148]. IRE1α mediates unconventional splicing of XBP1 (X-box binding protein 1) mRNA, enhancing expression of chaperones and ER-associated degradation (ERAD) components, but sustained IRE1α activation also induces JNK signaling, promoting oligodendrocyte apoptosis [149]. ATF6 translocates to the Golgi apparatus where it is cleaved and activated, subsequently upregulating genes involved in protein folding and quality control [150]. Persistent or unresolved ER stress disrupts myelin protein synthesis, thereby impairing myelin maintenance and repair, and may culminate in oligodendrocyte apoptosis, contributing to demyelination [151,152].

Myelin debris rich in immunogenic components, including MBP, MOG (myelin oligodendrocyte glycoprotein), and PLP, is produced by demyelination brought on by oligodendrocyte injury [152]. By stimulating microglial cells through TLR2 and TLR4 signaling and by making antigen presentation easier for peripheral immune cells, these molecules act as autoantigens, sustaining neuroinflammation [153]. The upregulation of co-stimulatory molecules (CD80/CD86) and the release of pro-inflammatory cytokines (e.g., IL-1β, TNF-α, and IL-6) are hallmarks of microglial activation in response to myelin debris, which exacerbates the local inflammatory milieu [154]. Moreover, it has been demonstrated that exosomes derived from oligodendrocytes that contain myelin antigens prime peripheral adaptive immune responses by promoting dendritic cell maturation and antigen presentation, thereby bridging the gap between systemic immunity and CNS inflammation [155].

Finally, oligodendrocytes express complement regulatory proteins like CD59; however, their expression is frequently downregulated during inflammatory stress, increasing the likelihood of complement-mediated lysis [156]. The membrane attack complex (MAC) is created when the complement cascade is activated, and it inserts into the oligodendrocyte membrane to cause cellular damage and death [157]. Complement activation products (e.g., C3a and C5a) serve as chemoattractants for infiltrating leukocytes and microglial cells, sustaining tissue damage and inflammation [158]. Another crucial factor in neuroinflammation is metabolic dysregulation in oligodendrocytes. Due to the upregulation of HIF-1α and its downstream glycolytic enzymes, such as pyruvate kinase M2 and hexokinase 2, numerous pro-inflammatory mediators cause a transition from oxidative phosphorylation to aerobic glycolysis [159]. Myelin formation and the maintenance of cellular integrity are compromised by this metabolic reprogramming, which is meant to meet energetic demands under stress but also causes lactate accumulation and reduced ATP production [160].

Oligodendrocyte-intrinsic susceptibilities, including complement-mediated cytotoxicity and metabolic reprogramming, are not isolated phenomena but are integrated within the broader glial network. Stressed or degenerating oligodendrocytes release cellular debris, pro-inflammatory cytokines, and metabolic by-products such as lactate, which function as paracrine signals modulating the activity of neighboring microglia and astrocytes. These oligodendrocyte-derived signals not only potentiate local inflammatory responses but also dynamically regulate glial activation states, establishing a self-perpetuating feed-forward loop that sustains and amplifies neuroinflammatory cascades.

### 3.4. Interplay Between Glial Populations

Conversely, the persistence of neuroinflammation depends on the interaction between glial populations. Cytokines, chemokines, extracellular vesicles, and metabolites like lactate and succinate all aid in microglial cells–astrocytes communication [161]. For example, succinate from activated microglial cells controls astrocyte reactivity by acting as a signaling molecule via SUCNR1 (succinate receptor 1) [162]. On the other hand, purinergic and metabotropic glutamate receptors allow astrocytic ATP and glutamate to affect microglial activation states [163,164]. In response to injury, oligodendrocytes (which are typically less interactive) secrete a variety of heat shock proteins and lipids, which are picked up by astrocytes and microglial cells and amplify the pro-inflammatory cascade [165,166]. The accumulation of oxidized lipids and misfolded proteins, such as α-synuclein, amyloid-β, and TDP-43 (TAR DNA-binding protein 43) aggregates, activates several glial pattern recognition receptors (PRRs), perpetuating chronic neuroinflammation and promoting neurodegenerative processes [167].

### 3.5. Additional Insights into Neuroinflammation Involving Glial Cells

The persistent activation of glial cells contributes to a maladaptive inflammatory environment that undermines neuronal integrity and function. Dysregulation of resolution pathways, including impaired production of anti-inflammatory cytokines (e.g., IL-10 and TGF-β), failure of lipid mediators such as resolvins and lipoxins, and inadequate clearance of apoptotic cells and debris through efferocytosis, prolongs glial activation and fosters a feed-forward loop of neuroinflammation [168,169]. These processes are vital to the origin and progression of some neurodegenerative and demyelinating conditions, highlighting the need for a deeper understanding of the molecular intricacies governing glial activation as a basis for developing targeted immunomodulatory therapies.

**Figure 3:**
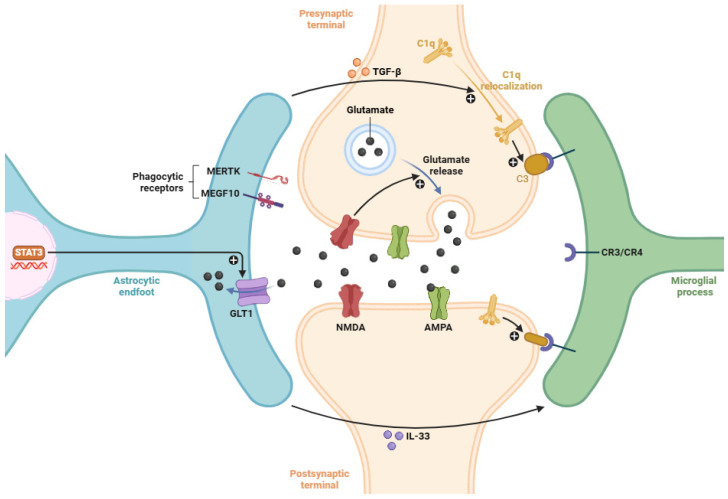
Diagram illustrating the mechanism of synaptic pruning in the CNS. Synaptic pruning is a key process in the developing and adult brain where excess or weak synapses are eliminated to refine neural circuits. In this mechanism, glutamate is released from the presynaptic terminal and binds to NMDA and AMPA receptors on the postsynaptic neuron, facilitating neurotransmission. Astrocytes maintain synaptic homeostasis by clearing excess glutamate via the GLT1 transporter. Meanwhile, signals like TGF-β from the presynaptic terminal induce the expression and relocalization of complement protein C1q, which, along with C3, tags synapses for elimination. These tags are recognized by CR3/CR4 receptors on microglial cells, which then engulf the marked synapses. Astrocytes also contribute to synaptic pruning through phagocytic receptors such as MERTK and MEGF10, regulated by the STAT3-SOCS3 pathway. Moreover, IL-33 released through astroglial end-feet further activates microglial pruning. The coordinated interplay of neurons, astrocytes, and microglial cells regulates synaptic remodeling during development, with microglial motility enabling interactions at neuron–astrocyte tripartite synapses. Abbreviations: STAT3 (signal transducer and activator of transcription 3), MERTK (MER tyrosine kinase), MEGF10 (multiple EGF-like domains 10), GLT1 (glutamate transporter 1), TGF-β (transforming growth factor beta), IL-33 (interleukin 33), C1q (complement Component 1q), NMDA (N-methyl-D-aspartate), AMPA (α-Amino-3-hydroxy-5-methyl-4-isoxazolepropionic acid), C3 (complement component 3), CR3 (complement receptor 3), and CR4 (complement receptor 4).

On the other hand, microglial cells and astrocytes play a key role in synaptic pruning (Figure 3), with several molecular pathways that intersect with neuroinflammatory processes [170]. Under normal developmental conditions, microglial cells mediate synaptic pruning through the complement cascade [171]. Neurons tag less active or unnecessary synapses with complement proteins (e.g., C3). Microglial cells express some complement receptors CR3 (CD11b/CD18) and CR4 (CD11c/CD18), which bind to synapses opsonized with complement fragments C3b or its inactivated form iC3b. This interaction allows the recognition, engulfment, and subsequent clearance of tagged synapses through receptor-mediated phagocytosis [172]. Astrocytes, despite their limited phagocytic capacity compared to microglial cells, contribute by secreting several cytokines (e.g., IL-33, TGF-β, and CXCL10) that modulate strongly microglial pruning activity [173]. Astrocytes also upregulate MEGF10 (MER tyrosine kinase) and MERTK (MER tyrosine kinase), two phagocytic receptors that mediate engulfment of synaptic elements under neuroinflammation [174].

In the context of neuroinflammation, microglial activation prompts the expression of complement components and phagocytic receptors [175]. This inflammatory state might lead to excessive or inappropriate synaptic pruning (Figure 3), a phenomenon implicated in disorders such as AD or MS [176,177]. Chronic inflammatory states are also associated with the depletion of several homeostatic markers (such as P2Y12 and TMEM119), favoring a pro-phagocytic phenotype that lacks the temporal and spatial precision documented during development [178]. This aberrant activation disrupts the balance of synaptic connectivity, contributing to cognitive decline and behavioral deficits associated with neuroinflammation [179].

Throughout chronic neuroinflammation, microglial activation extends beyond conventional immune functions, influencing neuronal circuitry and modulating astrocyte and oligodendrocyte activity through the secretion of several pro-inflammatory cytokines and metabolic mediators. This intercellular crosstalk establishes an intricate glial network that perpetuates neuroinflammatory responses and connects cellular dysfunction to CNS deficits, offering a conceptual framework for the study of glial interactions in neurodegenerative disorders.

## 4. Interplay Between Glial Cells, Neuroinflammation, and CNS Disorders

Important mediators of neuroinflammation, glial cells are increasingly recognized as context-dependent regulators of CNS homeostasis whose roles vary across disease stages and pathological environments [9]. While their early responses are often protective, persistent activation driven by disease-specific triggers and genetic susceptibility can adopt glial phenotypes toward maladaptive, self-sustaining inflammatory states, ultimately exacerbating neuronal dysfunction and degeneration [180].

In MS, glial responses are tightly coupled to autoimmune initiation and relapse-remission dynamics, with microglial cells and astrocytes acting as key amplifiers of peripheral immune infiltration rather than primary initiators of pathology. TLRs and cytokine receptors (TNFR and IFNGR) promote early microglial activation, leading to ROS, RNS, and NO production via NADPH oxidase and subsequent oxidative injury to myelin and axons [181]. As a disease progresses, sustained NLRP3 inflammasome activation reinforces chronic lesion activity, driving the release of IL-1β, IL-6, and TNF-α [182]. In parallel, microglia enhance antigen presentation via upregulation of MHC-II and CD86, thus linking innate immune activation to adaptive autoimmune responses [183]. Astrocytes can contribute in a disease-stage-dependent manner by chemokines (CCL2 and CXCL10), VEGF, and MMP-2/9, which facilitate leukocyte recruitment and blood–brain barrier disruption during active lesions [184]. Astrocytic glutamate dysregulation becomes more prominent during chronic stages, worsening excitotoxicity [185]. Oligodendrocyte apoptosis, driven by oxidative stress and immune-mediated caspase-3 activation, leads to progressive myelin protein loss and limits remyelination capacity, a hallmark of progressive MS [186].

In AD, glial activation evolves from an early protective clearance response toward a persistent maladaptive phenotype driven by aging-related factors, APOE genotype, and metabolic stress. Microglia recognize amyloid-β through TLR2/4, CD36, and TREM2, triggering prolonged NLRP3 inflammasome activation and sustained IL-1β and IL-18 release [187,188]. TREM2-dependent signaling initially supports plaque containment, but genetic variants impair this transition and favor neurotoxic inflammation. Persistent NOX2-mediated ROS and NO production further potentiate oxidative stress and compromises amyloid clearance [188]. Astrocytes undergo NF-κB-dependent reactive astrogliosis, releasing IL-6, TNF-α, and S100B, which contributes to synaptic dysfunction and neuronal apoptosis rather than acute cell loss [189]. Metabolic uncoupling becomes increasingly relevant at later stages, as reduced lactate delivery and impaired glutamate uptake undermine synaptic resilience and accelerate cognitive decline [190,191].

Neuroinflammation in ALS is closely linked to non-cell-autonomous mechanisms, in which glial dysfunction critically shapes motor neuron vulnerability. Microglia adopt a persistent pro-inflammatory M1-like phenotype, with NF-κB and MAPK activation driving secretion of IL-1β, TNF-α, NO, and prostaglandins [192,193]. This inflammatory profile intensifies with disease progression and is modulated by genetic factors such as SOD1 and C9orf72 mutations. Astrocytes progressively lose neuroprotective functions, leading to EAAT2 downregulation and glutamate-driven excitotoxicity. Moreover, astrocytes secrete neurotoxic mediators including prostaglandin E2, ROS, and TGF-β, contributing to selective motor neuron degeneration [194]. Oligodendrocytes further exacerbate pathology by reducing MCT1 expression, limiting lactate supply and compromising long-range axonal integrity during later disease stages [195].

Finally, in PD, glia-mediated oxidative and inflammatory cascades are critically influenced by environmental toxic exposures, intrinsic mitochondrial susceptibility, and α-synuclein-driven pathology. Misfolded α-synuclein activates microglial cells via TLR2/4 and NLRP3, inducing sustained ROS, TNF-α, and IL-1β production [196]. Dopaminergic neurons in the substantia nigra pars compacta are susceptible due to high basal oxidative load and NOX2-dependent microglial bursts, compounded by mitochondrial dysfunction [197]. Astrocytic dysfunction further exacerbates degeneration through reduced EAAT2-mediated glutamate uptake and impaired glutathione synthesis, compromising antioxidant defenses [198]. Loss of astrocyte-derived trophic support (influenced by GDNF and BDNF) precedes overt neuronal loss, while reactive astrocytes produce and secrete MMP-9, IL-6, and CXCL1, sustaining chronic inflammation and synaptic instability [199].

Overall, glial cells orchestrate neuroinflammatory and neurodegenerative processes in a disease-specific and temporally dynamic manner, via glial crosstalk shaping neuronal vulnerability and progression, thus highlighting glial phenotypic modulation as a promising therapeutic strategy.

## 5. Therapeutic Targeting of Glial Cells

Targeting glial cells has emerged as a compelling and multifaceted therapeutic strategy in the context of CNS disorders [38]. Previously viewed as ancillary to neuronal function, glial cells are now recognized as critical regulators of neuroinflammatory processes, synaptic function, myelination, and cerebral metabolism. These functional domains represent key nodes of vulnerability and opportunity across a wide spectrum of neurological diseases, such as neurodegenerative, neurodevelopmental, and neuroinflammatory conditions.

Their involvement in both the initiation and resolution of inflammation, in modulating synaptic connectivity and plasticity, and in maintaining metabolic and redox homeostasis, positions glial cells as attractive and versatile therapeutic targets. Importantly, glial dysfunction is increasingly implicated as a primary driver, rather than a secondary consequence, of CNS pathology. This has prompted a reorientation of drug development efforts toward cell-type-specific interventions aimed at modulating glial activity and phenotype.

Early trials aimed at modulating glial activity have yielded promising results, showing favorable effects on inflammatory biomarkers and, in some phase I–II studies, modest indications of clinical efficacy. However, larger-scale trials have often produced negative or inconsistent outcomes, and important safety concerns remain. Importantly, central inflammatory responses and imaging-detected side events (e.g., edema or microhemorrhages), reminiscent of amyloid-related imaging abnormalities (ARIA) reported with anti-amyloid antibodies, underscore the potential risks associated with broad modulation of immune functions in the CNS [200]. Additionally, substantial translational gaps remain, as preclinical models fail to recapitulate the cellular heterogeneity and complexity of the human glial network and do not account for the influence of aging and comorbidities [201]. Therapeutic windows and optimal dosing might differ across species, whereas existing several biomarkers often limited reliability in forecasting long-term clinical outcomes [202]. These limitations underscore the need for caution in translating glia-targeted strategies to the clinic, reinforcing the need of controlled, biomarker-guided experimental designs, cohort selection, rigorous imaging and biochemical monitoring, and stepwise dose escalation before broader implementation is considered [203]. Such approaches will help to mitigate risks and bridge the gap between preclinical insights and reproducible clinical benefit.

This section critically examines the evolving landscape of therapeutic approaches directed at glial cell populations. An overview is provided of current anti-inflammatory strategies targeting glial-mediated neuroinflammation, highlight the identification of glia-specific molecular targets and signaling pathways, and assess the potential of emerging modalities such as cell-based therapies, gene editing technologies, and glial reprogramming. On the other hand, the application of nanomedicine for the targeted delivery of pharmacological agents across the BBB is examined, with particular emphasis on those technologies that strengthen cellular specificity, minimize systemic adverse effects, and enable precise engagement of glial cells. Collectively, these approaches reflect a growing recognition of glial cells as central players in CNS homeostasis and dysfunction, and as key leverage points for next-generation therapeutic interventions.

### 5.1. Current Anti-Inflammatory Strategies

The majority of CNS disorders are characterized by the inflammatory response that is coordinated by glial cells, especially microglial cells and astrocytes. Chronic glial activation causes the sustained release of several pro-inflammatory cytokines (such as TNF-α, IL-1β, and IL-6), ROS, and excitotoxic agents like glutamate, which contribute to neuronal injury, even though acute inflammation can be protective.

#### 5.1.1. Non-Steroidal Anti-Inflammatory Drugs (NSAIDs)

The potential of non-steroidal anti-inflammatory drugs (NSAIDs; Table 1), such as ibuprofen and indomethacin, to modify glial-mediated inflammation has been thoroughly studied, especially in relation to neurodegenerative diseases like AD, MS, and ALS [204,205,206,207,208,209,210,211,212]. These drugs mainly work by blocking the cyclooxygenase (COX) enzymes, namely COX-1 and COX-2, which catalyze the production of prostaglandins from arachidonic acid [213]. In the CNS, COX-2 is primarily expressed in neurons and, in pathological situations, in activated microglial cells and astrocytes [214]. Increased synthesis of pro-inflammatory prostaglandins like PGE_2_, which amplify glial activation, enhance cytokine production (e.g., IL-1β and TNF-α), and disrupt neuronal function and survival, is caused by the upregulation of COX-2 in neuroinflammatory states [215]. NSAIDs decrease prostaglandin levels by blocking COX-2, which can lessen excitotoxicity, oxidative stress, and glial activation [216].

Furthermore, it has been demonstrated that several NSAIDs have COX-independent effects, which could be a factor in their neuroprotective qualities [217]. One nuclear receptor implicated in anti-inflammatory signaling, peroxisome proliferator-activated receptor gamma (PPARγ), has been shown to be activated by certain NSAIDs [218]. A key pathway in the transcription of pro-inflammatory genes in microglial cells and astrocytes, NF-κB signaling, is downregulated when PPARγ is activated [219].

The clinical application of NSAIDs for neurodegenerative diseases has been mainly disappointing, despite these encouraging mechanisms seen in certain preclinical models. Their poor permeability across the BBB is a significant drawback, leading to subtherapeutic levels in the CNS [220]. The therapeutic results of NSAIDs are further complicated by their lack of cell-specificity and potential to influence both beneficial and detrimental glial responses.

Celecoxib and other COX-2-selective inhibitors were created to more accurately target inflammation and reduce the gastric side effects linked to non-selective NSAIDs [221]. However, more research is needed to validate these findings, even though their effectiveness in some clinical trials for AD has shown promise [222,223].

**Table 1:** Table illustrating various anti-inflammatory therapies that inhibit glial cell overactivation, including their drug family, specific compounds, molecular mechanisms of action, and associated side effects. Abbreviations: NSAID (nonsteroidal anti-inflammatory drug), COX-1 (cyclooxygenase 1), COX-2 (cyclooxygenase 2), PML (progressive multifocal leukoencephalopathy), VCAM-1 (vascular cell adhesion molecule 1), TNF-α (tumor necrosis factor-alpha), IL-1β (interleukin 1 beta), IL-6R (interleukin 6 receptor), Aβ (amyloid beta), and ARIA (amyloid-related imaging abnormalities).

Drug Family	Compound	Molecular Mechanisms	Key Strengths	Side Effects	References
NSAIDs	Ibuprofen Indomethacin Celecoxib	Inhibition of COX-1 and COX-2 Activation of PPARγ	Widely available Rapid anti-inflammatory effect	Gastrointestinal bleeding Renal dysfunction Cardiovascular risk Hypersensitivity reactions Headache Dizziness Renal toxicity	[213,214,215,216,218,219,224]
Minocycline		Reduction in cytokine production Modulation of apoptosis	BBB penetration Neuroprotective potential	Dizziness Skin pigmentation Autoimmune hepatitis Hypersensitivity syndrome	[225,226,227,228,229,230]
mAbs	Natalizumab	Blocks α4 integrin-VCAM-1 interactions	High efficacy in immune-mediated CNS diseases	PML Hypersensitivity reactions Risk of infections	[231,232,233]
	Infliximab Adalimumab	Neutralization of TNF-α	Strong systemic anti-inflammatory effects	Risk of infections Malignancies Autoimmune responses	[234,235,236,237,238,239,240]
	Canakinumab	Blockade of IL-1β	Targeted cytokine suppression	Risk of infections Neutropenia Gastrointestinal symptoms	[241,242,243,244]
	Tocilizumab	Antagonizes IL-6R		Serum transaminase elevation Risk of infections Gastrointestinal bleeding Neutropenia	[245,246]
	Aducanumab	Inhibits Aβ plaques formation	First approved disease- modifying AD therapy	ARIA Headache Confusion Falls	[247,248,249,250]

#### 5.1.2. Minocycline

Using molecular mechanisms that alter cellular signaling pathways, the second-generation tetracycline antibiotic minocycline (Table 1) exhibits a pharmacological profile that goes well beyond its traditional antimicrobial activity. It also demonstrates notable anti-inflammatory and anti-apoptotic effects [225]. Minocycline mainly inhibits microglial activation, a major cause of neuroinflammation in many CNS pathologies, to produce neuroprotective effects [226]. The suppression of NF-κB and MAPK pathways causes this inhibition, which in turn reduces transcription and the release of many pro-inflammatory cytokines, including TNF-α, IL-1β, and IL-6 [227,228].

Moreover, minocycline reduces programmed cell death in susceptible neurons by inhibiting mitochondrial cytochrome c release and subsequent caspase-3 activation, which modulates apoptotic cascades [229]. These molecular actions result in reduced lesion size, reduced BBB disruption, and preserved neuronal architecture in preclinical models of ischemic stroke, spinal cord injury, and autoimmune neuroinflammatory diseases such as MS. These outcomes all contribute to improved behavioral and functional recovery [251,252,253,254]. Long-term use of minocycline has been associated with potential negative off-target effects, including mitochondrial dysfunction, pigmentary abnormalities, and the development of autoimmune responses, despite the drug’s significant therapeutic benefits [230]. Their interactions with different cellular components outside of the CNS are probably the cause of these side effects, which emphasizes the necessity of carefully evaluating dosage regimens and long-term safety profiles in clinical applications [255].

#### 5.1.3. Therapeutic Modulation of Cytokines: Experimental Methodologies

Monoclonal antibody (mAb) therapies have emerged as a transformative strategy in treating neuroinflammatory disorders due to their specificity in targeting pathological molecular pathways within the CNS (Table 1) [256]. A prominent example is natalizumab, an anti-α4 integrin antibody that inhibits leukocyte adhesion to VCAM-1, thus preventing immune cell migration across the BBB, a crucial mechanism in MS pathogenesis [231]. By preventing peripheral immune cell infiltration into the CNS, natalizumab modulates the neuroinflammatory milieu, resulting in decreased activation of microglial cells and astrocytes and attenuation of associated pro-inflammatory signaling pathways [232].

On the other hand, mAbs targeting TNF-α, such as infliximab and adalimumab, neutralize both soluble and membrane-bound forms of TNF-α, preventing downstream pro-inflammatory signaling [234,235]. By blocking TNF-α activity, these antibodies contribute to the complete suppression of glial activation and help mitigate the chronic neuroinflammatory responses associated with several neurodegenerative and autoimmune conditions [234,236,257]. However, these drugs compromise immunosurveillance, thereby increasing susceptibility to opportunistic infections [237,238,239,240]. Other cytokine-targeting mAbs include canakinumab, which blocks IL-1β, reducing the production of downstream mediators like IL-6 and MMPs [241,242,243,244], and tocilizumab, which antagonizes IL-6 receptor (IL-6R) signaling to dampen CNS inflammation [245,246].

In AD, therapies such as aducanumab target amyloid-beta (Aβ) plaques, promoting their clearance via microglial phagocytosis and reducing associated neuroinflammation and synaptic damage [247,248,249,250]. Finally, combination therapies targeting multiple inflammatory mediators are under evaluation to improve outcomes in some neuroinflammatory diseases (e.g., the combination of natalizumab—anti-α4 integrin, alemtuzumab—anti-CD52, ocrelizumab—anti-CD20, and daclizumab—anti-CD25 in MS [258]).

### 5.2. Glia-Specific Pharmacological Targets

Emerging insights into glial biology have identified some molecular targets uniquely or preferentially expressed by glial cells, opening the door to glia-specific pharmacological interventions, which hold the potential to modulate neuroinflammatory and neurodegenerative processes with greater precision while minimizing off-target effects in neurons and other CNS cell types, although their translational efficacy remains to be validated.

#### 5.2.1. Microglial Targets

Colony-Stimulating Factor 1 Receptor (CSF1R). CSF1R is a class III receptor tyrosine kinase expressed on microglial cells and other phagocytes [259]. Activation by CSF1 or IL-34 induces receptor dimerization and autophosphorylation, triggering intracellular signaling cascades such as PI3K-AKT, MAPK/ERK, and JAK/STAT, which control microglial survival, proliferation, and differentiation [260]. Several small-molecule inhibitors (e.g., PLX3397 and GW2580) block the ATP-binding domain of CSF1R, leading to microglial depletion in numerous preclinical models [261,262]. These agents ameliorate neuroinflammation and neurodegeneration in preclinical models of AD and PD by suppressing microglial activation [263,264,265]. However, uninterrupted depletion might impair key microglial functions, like synaptic pruning, debris clearance, and neurogenesis, highlighting the therapeutic relevance of selective modulation over complete ablation (Table 2) [266].

Triggering Receptor Expressed on Myeloid Cells 2 (TREM2). TREM2 is a microglial surface receptor that signals via the adaptor DAP12 (DNAX activating protein of 12 kDa), containing ITAM (immunoreceptor tyrosine-based activation motif) motifs [267]. Ligand binding (e.g., lipids and ApoE -Apolipoprotein E-) induces DAP12 phosphorylation and activation of Syk (spleen tyrosine kinase), initiating downstream signaling via PI3K-AKT, PLCγ (phospholipase C gamma), and mTOR (mechanistic target of rapamycin) pathways [268]. TREM2 enhances phagocytosis, lipid metabolism, and cell survival, and facilitates the transition to a DAM phenotype [269]. Agonistic mAbs (e.g., AL002) that potentiate TREM2 signaling are in clinical development, aiming to promote amyloid-β clearance and shift microglial cells toward a protective, anti-inflammatory state (Table 2) [270].

**Table 2:** Summary of therapeutic strategies targeting glial cell hyperactivity: glial and molecular targets, pharmacological compounds, biological effects, and implications in some CNS pathologies. Abbreviations: CSF1R (colony-stimulating factor 1 receptor), ATP (adenosine triphosphate), TREM2 (triggering receptor expressed on myeloid cells 2), AD (Alzheimer’s disease), PD (Parkinson’s disease), AQP4 (aquaporin 4), NMOSD (neuromyelitis optica spectrum disorder), MCAO (middle cerebral artery occlusion), CCI (chronic constriction injury), CIPN (chemotherapy-induced peripheral neuropathy), Cx43 (connexin 43), EAAT2/GLT-1 (excitatory amino acid transporter 2/glutamate transporter 1), CNS (central nervous system), EAE (experimental autoimmune encephalomyelitis), MAI (myelin-associated inhibitor), LINGO-1 (leucine-rich repeat and Ig domain-containing Nogo receptor-interacting protein 1), and Nogo-A (neurite outgrowth inhibitor A).

Glial Target	Molecular Target	Compounds	Biological Effects	CNS Pathology	References
Microglial cells	CSF1R	PLX3397 GW2580	Reduction in neuroinflammation and neurodegeneration by inhibiting the ATP-binding site of CSF1R, which suppresses chronic microglial activation and induces microglial depletion	Preclinical studies: Mouse AD (in vivo experiments)	[263]
				Preclinical studies: Rat PD (in vivo experiments)	[264]
				Preclinical studies: Mouse AD (in vivo experiments)	[265]
	TREM2	AL002	AL002, currently in clinical development, aims to enhance amyloid-β clearance and promote a protective microglial phenotype	Clinical studies: Clinical trial in AD patients	[266]
Astrocytes	AQP4	Anti-AQP4 mAb	Reduction in neuroinflammation and neurodegeneration	Preclinical studies: NMOSD rodents (in vitro and in vivo experiments)	[271]
		Arbidol Tamarixetin		Preclinical studies: NMOSD mouse (in vitro and in vivo experiments)	[272]
	Cx43	Gap19		Preclinical studies: MCAO mouse (in vitro and in vivo experiments)	[273]
		Peptide5		Preclinical studies: CCI and CIPN mouse (in vitro and in vivo experiments)	[274]
		RNAi		Preclinical studies: Various in vitro and in vivo experiments in injured rodents	[275]
	EAAT2/GLT-1	Ceftriaxone	EAAT2/GLT-1 upregulation restores CNS homeostasis and mitigates neuroinflammation and neurodegeneration	Preclinical studies: EAE mouse (in vitro and in vivo experiments)	[276]
		LDN/OSU-0212320		Preclinical studies: Various in vitro and in vivo experiments in injured rodents	[277]
		Riluzole		Preclinical studies: EAE mouse (in vitro and in vivo experiments)	[278]
Oligodendrocytes	MAI	Anti-LINGO-1 Anti-Nogo-A	Support the maturation of oligodendrocytes and repair of several demyelinated areas	Preclinical studies: Various in vitro and in vivo experiments in injured rodents	[279]

#### 5.2.2. Astrocytic Targets

Aquaporin-4 (AQP4). It is the principal water channel in the CNS, localized predominantly at astrocytic end-feet through interactions with the dystrophin-associated protein complex (DAPC), including α-syntrophin, dystrophin (Dp71), and agrin [280]. This polarization is vital for regulating water flux across the BBB [281]. AQP4 mediates bidirectional water transport in response to osmotic gradients and is regulated transcriptionally and post-translationally (e.g., phosphorylation at Ser111, Thr157, and Ser180; ubiquitination) [282,283]. In CNS injury (e.g., stroke and trauma), AQP4 allows cytotoxic edema through astrocytic water influx and later contributes to vasogenic edema clearance [284]. Furthermore, AQP4 modulates neuroinflammation through cytokine signaling (such as IL-1β and TNF-α via NF-κB) and is itself regulated by STAT3-dependent signaling during reactive astrogliosis [285]. In neuromyelitis optica spectrum disorder (NMOSD), AQP4 is the main target of pathogenic IgG1 antibodies, inducing complement- and antibody-dependent cytotoxicity [286]. Therapeutic strategies (Table 2) include anti-AQP4 mAbs and some small-molecule inhibitors (e.g., arbidol and tamarixetin) [271,272].

Connexin43 (Cx43), encoded by GJA1 gene (gap junction alpha 1), forms astrocytic gap junctions via hexameric connexons, supporting intercellular exchange of ions and small metabolites for homeostatic functions [273]. While gap junctions remain open, hemichannels are typically closed but become pathologically activated by injury, ischemia, or inflammation, leading to ATP and glutamate release and promoting inflammasome activation (e.g., via P2X7 receptors) [287]. Cx43 function is modulated by phosphorylation (PKC, ERK1/2, CK1—casein kinase 1, and Src—proto-oncogene tyrosine-protein kinase Src), influencing assembly and gating [288]. Hemichannel blockade (using agents like Gap19) reduces neuroinflammation and secondary damage in models of stroke and epilepsy [289]. Other tactics include mimetic peptides (e.g., Peptide5) and RNAi (RNA interference; Table 2) [274,275].

Glutamate Transporters (EAAT2/GLT-1) are high-affinity, Na^+^-dependent transporters localized on perisynaptic astrocyte membranes, responsible for >90% of extracellular glutamate clearance [290]. EAAT2 activity is electrogenic, coupling glutamate uptake with Na^+^/H^+^ influx and K^+^ efflux [291]. Its expression is highly regulated via NF-κB and CREB (cAMP response element-binding) pathways [292]. Under inflammation, EAAT2 is downregulated through epigenetic and proteasomal mechanisms, contributing to excitotoxicity [293]. EAAT2 dysfunction is strongly associated with ALS, AD, HD, and epilepsy [294,295,296,297]. Therapeutic upregulation strategies include β-lactams (e.g., ceftriaxone), small-molecule enhancers (e.g., LDN/OSU-0212320), and riluzole (Table 2) [276,277,278].

#### 5.2.3. Oligodendroglial Targets

Myelin-associated inhibitors (MAIs), like Nogo-A and LINGO-1, negatively regulate remyelination in the CNS by activating the Nogo receptor 1 (NgR1) with co-receptors such as p75^NTR^, TROY (tumor necrosis factor receptor superfamily member 19), and LINGO-1 [298]. This activation triggers the RhoA (Ras homolog family member A)/ROCK (Rho-associated coiled-coil-containing protein kinase) signaling pathway, which inhibits cytoskeletal remodeling necessary for axonal growth and OPC differentiation [279]. LINGO-1 acts as a crucial co-receptor mediating suppression of OPC maturation [299]. Therapeutic agents targeting Nogo-A or LINGO-1 aim to promote oligodendrocyte differentiation and myelin repair, particularly in MS (Table 2) [279].

Monocarboxylate transporter 1 (MCT1), mainly expressed on oligodendrocytes, mediates the transport of lactate and pyruvate, encouraging metabolic coupling between oligodendrocytes and neurons [300]. Oligodendrocytes convert glucose to lactate, which is shuttled through MCT1 to axons, where neurons uptake it through MCT2 (monocarboxylate transporter 2) to support mitochondrial energy production [301]. Impaired MCT1 function in those demyelinating diseases disrupts metabolic coupling between oligodendrocytes and neurons, leading to energy deficits and degeneration [302]. Improving MCT1 activity constitutes a potential therapeutic strategy to restore neuronal metabolic support and prevent axonal loss in MS [303].

### 5.3. Gene Therapies

Innovative strategies involving gene editing technologies are emerging as powerful tools with unprecedented potential to reprogram dysfunctional glial populations. These approaches not only enable targeted manipulation of glial cell identity and function, but also open new avenues for treating a wide range of neurodegenerative and neurodevelopmental disorders where glial dysfunction plays a key role [304].

#### 5.3.1. Viral Vector-Based Gene Delivery

Adeno-associated viruses (AAVs) have emerged as the vectors of choice for in vivo gene delivery due to their low immunogenicity, long-term expression, and capacity for tissue-specific targeting (Table 3) [305]. Recent findings in AAV engineering have enabled the use of glial-specific promoters (e.g., GFAP, Iba1—ionized calcium binding adapter molecule 1, and MBP) to drive selective expression of therapeutic genes in specific glial subpopulations [306,307]. This precision targeting is valuable for modulating the glial contribution to neurodegenerative and neuroinflammatory pathologies. For instance, AAV-mediated overexpression of neuroprotective molecules including IGF-1 (insulin-like growth factor 1) or BDNF in astrocytes have been shown to confer neuroprotection, reduce excitotoxicity, and support synaptic integrity [308,309]. Concurrently, the delivery of genetic material to microglial cells through tailored AAV vectors shift these cells toward a homeostatic, anti-inflammatory phenotype, thus mitigating chronic neuroinflammation and associated neurodegeneration [310]. Current studies aim to optimize vector serotypes and promoter designs aimed at improving gene transfer efficiency, target-cell specificity, and biosafety, with the aim of enhancing their applicability to translational medicine [311].

#### 5.3.2. RNA-Based Therapies

Small interfering RNAs (siRNAs) and antisense oligonucleotides (ASOs) are two examples of RNA (ribonucleic acid)-targeting modalities that provide tools for post-transcriptional modification of gene expression in glial cells (Table 3). These molecules can be designed to suppress the translation of deleterious proteins, restore normal splicing patterns, or selectively target and degrade mutant transcripts [312,313]. ASOs (targeting Sod1 -superoxide dismutase 1- and/or C9orf72) transcripts have been developed in the context of ALS to lessen the buildup of toxic proteins and RNA foci in neurons, astrocytes, and microglial cells, which contribute to the progression of the disease [314,315]. ASOs are especially well-suited for repeated therapeutic administration due to their stability and capacity to cross the BBB [316]. Moreover, glial-derived inflammatory mediators and oxidative stress-related enzymes have been demonstrated to be downregulated by siRNAs [317]. With many RNA-based treatments currently making their way via clinical trials for different CNS disorders, these therapeutic options represent a rapidly developing frontier in glia-targeted therapy.

### 5.4. Cell Replacement Therapy

#### 5.4.1. Astrocyte Transplantation

The transplantation of astrocyte precursors has emerged as a promising therapeutic approach in models of neurodegenerative and traumatic CNS disorders, mainly SCI and ALS (Table 4) [318,319]. Preclinical studies have shown that the introduction of astrocyte precursor cells into the injured spinal cord improves neuronal viability, reduces gliosis, and allows axonal regeneration [320,321]. An important advancement in this field is the derivation of astrocytes from human induced pluripotent stem cells (iPSCs), which are currently under investigation for their safety and efficacy in several clinical settings [322]. These iPSC-derived astrocytes not only exhibit functional properties consistent with endogenous astrocytes but also hold the potential for personalized, autologous cell therapy strategies [323].

#### 5.4.2. OPC Transplantation

OPC transplantation represents a novel strategy to restore myelination in several demyelinating diseases including MS and in the context of traumatic SCI (Table 4) [324]. In preclinical models, transplanted OPCs have been shown to migrate to those demyelinated regions, differentiate into mature oligodendrocytes, and effectively remyelinate axons [325,326]. This remyelination contributes to the restoration of nerve conduction velocities and amelioration of functional deficits [327]. iPSC-derived OPCs have shown promising results in terms of scalability, reproducibility, and potential for clinical translation [328].

#### 5.4.3. Microglial Transplantation

Microglial replacement therapy is an emerging experimental approach intended for reestablishing a balanced neuroimmune environment within the CNS (Table 4). Dysregulation of microglial function is implicated in many neurological disorders, such as neurodegenerative diseases and CNS injuries. Some therapeutic strategies for replacing dysfunctional microglial cells include the transplantation of hematopoietic stem cells, which can differentiate into microglia-like cells within the CNS, as well as the transplantation of microglia derived from human iPSCs, offering a targeted and physiologically relevant approach to restoring microglial function [329].

Recent studies using mouse models with human CSF1R mutations demonstrated that microglial depletion followed by replacement with healthy bone marrow-derived microglial cells (Mr BMT) restored myelin integrity, reduced axonal spheroids, and improved cognitive function. This approach was applied to eight ALSP (adult-onset leukoencephalopathy with axonal spheroids and pigmented glia) patients using conventional bone marrow transplantation, resulting in stabilization of disease progression over a 24-month period [330]. Moreover, direct intracerebral injection of Sca1^−^ (stem cell antigen-1 negative) myeloid progenitor cells has been shown to efficiently repopulate the microglial compartment in the absence of myeloablative preconditioning, thus providing proof-of-concept for microglial replacement. This approach rescued a mouse model of Sandhoff disease and highlighted the potential of human iPSC-derived progenitors as an alternative to conventional hematopoietic stem cell transplantation [331]. The replacement of dysfunctional microglial cells with peripherally derived microglia-like cells (MLCs) renews CNS homeostasis, constituting an alternative therapeutic strategy for neurodegenerative pathologies associated with GM2 (GM2 ganglioside) accumulation, including Sandhoff disease [332].

While these interventions are still in early stages of development, preliminary studies suggest that appropriately engrafted microglial cells can integrate into the CNS tissues, assume homeostatic phenotypes, and modulate several inflammatory responses [333]. Further investigations are required to optimize delivery methods, ensure long-term engraftment, and assess therapeutic efficacy in clinical settings [334].

### 5.5. Nanomedicine and Targeted Drug Delivery

The BBB continues to represent a major impediment to the effective delivery of therapeutic agents to CNS [335]. Its highly selective permeability restricts the passage of most pharmacological compounds, thereby limiting treatment efficacy for numerous neurological disorders. In this context, the field of nanotechnology offers a transformative strategy to overcome the BBB’s restrictive properties [336]. Nanocarriers can be engineered to traverse the barrier via receptor-mediated transport or other mechanisms, enabling precise delivery of therapeutic payloads [337]. Additionally, these nanosystems (Table 5) offer the potential for cell-type-specific targeting, like selective delivery to glial cells, thus enhancing treatment specificity while minimizing off-target effects [338].

Polymeric nanoparticles (PNPs) and liposomes are effective nanocarriers that can encapsulate a variety of therapeutic agents, such as peptides, nucleic acids (such as plasmid DNA, siRNA, and miRNA—microRNA), and anti-inflammatory medications [339]. Targeting ligands like transferrin or the rabies virus glycoprotein (RVG) peptide can be added to the surface of these nanocarriers to functionalize them. These ligands attach to the receptors that are widely expressed on the BBB’s endothelial cells, like the transferrin receptor or the nicotinic acetylcholine receptor [340,341]. This makes it easier for the nanoparticles to cross the blood–brain barrier through receptor-mediated transcytosis. Through ligand-receptor interactions with glial cell markers (e.g., CD44 for astrocytes and CD11b for microglial cells), other cell-specific targeting can be accomplished after infiltrating the CNS parenchyma [342,343]. Therapeutic agents are released into the intracellular compartment upon cellular uptake, usually by clathrin or caveolin-mediated endocytosis. By disrupting pathways like NF-κB, JAK/STAT, or MAPK, these agents can exert gene-modulatory or anti-inflammatory effects [344,345].

Extracellular vesicles (EVs), such as exosomes, especially those originating from mesenchymal stem cells (MSCs) or engineered cell lines, have the innate ability to cross the BBB through endogenous pathways like adsorptive-mediated transcytosis or integrin-mediated adhesion and uptake [346]. Glial cells can more effectively recognize and internalize them due to their membrane biochemical composition, which is enriched in tetraspanins (such as CD63 and CD81) and integrins [347]. Through electroporation or endogenous sorting signals (e.g., EXOmotifs or exosomal motifs), engineered exosomes can be loaded with therapeutic proteins or regulatory miRNAs (like miR-124-3p) [348,349,350]. The exosomal contents, once internalized by glial cells, act on intracellular signaling cascades such as TLR4/NF-κB to influence neuroinflammatory signaling, promote neuroprotection, or rewire glial phenotypes [351]. Their immunological tolerance and physiological origin enable safe, repeated, and long-term therapeutic application in neurological disorders [352].

On the other hand, the BrainShuttle^TM^ platform employs engineered antibodies that bind to receptors on BBB endothelial cells, such as the transferrin receptor, and are actively transported into the CNS through receptor-mediated transcytosis. Following translocation across the BBB, therapeutic agents can interact with microglial cells and regulate their inflammatory signaling pathways [353]. Moreover, nanobodies (small antibody fragments derived from camelid antibodies) are advantageous due to their high stability, small size, and ability to recognize unique epitopes [354]. Nanobodies might represent an excellent strategy to target various molecules expressed in glial cells, aiming to enhance their functionality in the context of neuroinflammation [355].

Finally, Zhu et al. showed that topoisomerase 1 (TOP1) inhibitors, such as camptothecin and topotecan, suppress microglial and macrophage inflammation in vitro and reduce neuroinflammation in vivo. Furthermore, a β-glucan-coated DNA origami nanocarrier (TopoGami) was developed to deliver topotecan selectively to myeloid cells. This intervention suppressed microglial activation and attenuated disease progression in a MS-like model, underscoring the therapeutic potential of targeted TOP1 inhibition in neuroinflammatory disorders [356].

## 6. Conclusions

Beyond their historically assigned supporting roles in the CNS, glial cells are recognized as important regulators of neuroinflammation. While a substantial body of experimental work has clarified many aspects of glial activation, intercellular communication, and their involvement in neuropathological conditions, the strength and generalizability of this evidence vary considerably across model systems and pathological contexts. Much of the current mechanistic understanding derives from well-controlled animal models and in vitro studies, which have been instrumental in dissecting signaling pathways but do not always recapitulate the complexity and/or cellular diversity of the human CNS. Consequently, interpretations of glial-mediated processes must be framed with appropriate caution.

Glial intercellular communication constitutes a tightly regulated system that can promote tissue repair and homeostasis or, when dysregulated, contribute to neurodegeneration. However, the causal relationships between specific signaling pathways and defined pathological outcomes are often inferred rather than directly demonstrated, and their reproducibility across laboratories and disease models remains heterogeneous. In particular, context-dependent variables like species, developmental stage, brain region, and injury paradigm substantially influence observed outcomes.

Microglial activation encompasses a spectrum of phenotypic states ranging from pro-inflammatory and potentially neurotoxic responses to homeostatic and neuroprotective surveillance functions. On the other hand, reactive astrocytes adopt heterogeneous functional states that influence synaptic remodeling, BBB integrity, and neuronal survival. Although these concepts are supported by numerous transcriptomic and functional studies, the frequently cited phenotype classifications remain operational frameworks rather than universally validated biological entities, and their relevance to human disease is still under active investigation. These findings underscore the huge importance of conceptualizing glial plasticity, rather than relying on simplified or binary activation models.

Reciprocal interactions between microglia and astrocytes play a central role in shaping the neuroinflammatory cascade. Experimental evidence indicates that such interactions can amplify inflammatory signaling and sustain neuronal damage, but also facilitate debris clearance, trophic support, and inflammation resolution under specific regulatory conditions. Notably, the balance between these opposing outcomes is highly model-dependent, and direct evidence for analogous mechanisms operating in human neurodegenerative disorders remains limited. OPCs are increasingly recognized as immune-responsive cells capable of sensing pro-inflammatory mediators and modulating local immune environments; however, this emerging role is largely supported by preclinical studies and awaits rigorous validation in vivo and in human tissue.

The growing insights into glial biology have catalyzed a paradigm shift in CNS-focused therapeutic strategies. Targeting glial cells offers several opportunities to modulate immune regulation, metabolic homeostasis, and synaptic repair. Despite these advances, the clinical translation of glia-directed therapies has been constrained by issues like limited cell-type specificity, off-target effects, and insufficient consideration of the temporal and spatial dynamics of glial activation. Emerging approaches (e.g., glia-targeted molecular therapies, genome editing technologies, cell-based interventions, and nanotechnology-enabled delivery platforms) offer promising avenues, but most remain at early or preclinical stages of development. Importantly, claims regarding therapeutic efficacy should be interpreted cautiously until supported by reproducible, longitudinal, and clinically relevant data. The identification and robust validation of biomarkers capable of discriminating distinct glial states will be essential for advancing precision medicine approaches in CNS disorders [319], yet such biomarkers are currently scarce and incompletely validated.

Despite notable progress, significant limitations constrain the current understanding of glial biology. A large proportion of available evidence is derived from animal models that only partially reproduce the molecular, cellular, and clinical features of human CNS diseases. In vitro systems, while valuable for mechanistic dissection, often employ reductionist conditions that fail to capture the full diversity and plasticity of glial responses in vivo. Furthermore, technical variability, batch effects, and analytical challenges in single-cell and spatial transcriptomics complicate cross-study comparisons and limit reproducibility. These constraints highlight the need for longitudinal, cross-species, and integrative research frameworks capable of bridging mechanistic insights with clinical relevance.

Taken together, the literature supports several overarching principles in glial-driven neuroinflammation, while also revealing important gaps in knowledge. Neuroinflammatory processes should be viewed as emergent properties of coordinated multicellular glial networks rather than isolated and cell-autonomous responses. Glial activation is intrinsically context-dependent, influenced by temporal stage, regional identity, disease etiology, and systemic factors, pointing out the limitations of classification frameworks. Moreover, bidirectional communication between glial cells and neurons, as well as among different glial subtypes, represents a critical regulatory axis that determines whether inflammatory signaling culminates in repair or degeneration. Notably, the relative contribution of each component of this axis remains incompletely resolved in human disease settings.

**Figure 4:**
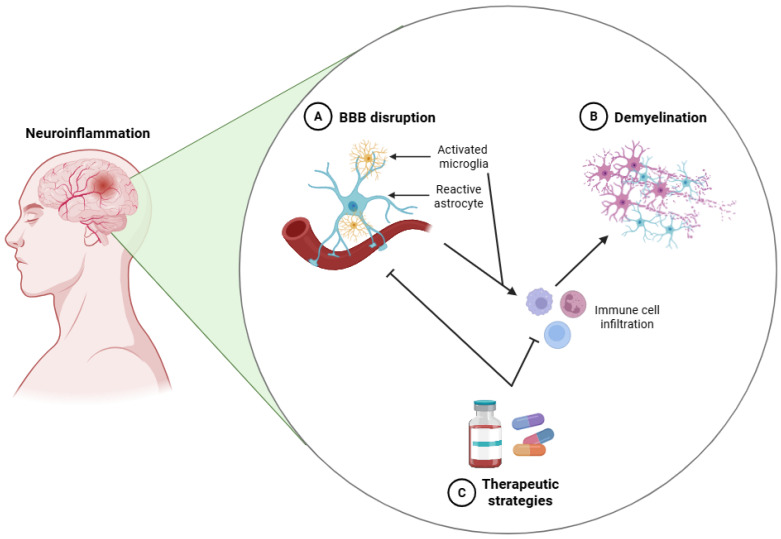
Neuroinflammatory mechanisms underlying BBB disruption: glial cell activation, immune cell infiltration, and subsequent demyelination, with emphasis on potential therapeutic interventions targeting these pathological processes. Abbreviations: BBB (blood–brain barrier).

In summary, glial cells (Figure 4) are key regulators of both the initiation and resolution of neuroinflammation, necessitating a shift in therapeutic strategies that recognizes them as active modulators of CNS immunity and plasticity. Future research should focus on comprehensive characterization of glial states across the lifespan and disease spectrum, delineation of conserved versus disease-specific signaling networks, and integration of multi-omic, spatial, and longitudinal data. Translational, systems-level studies will be critical to differentiate robust mechanisms from context-dependent observations and to fully realize the therapeutic potential of glial cells.

## Figures and Tables

**Table 3:** Glia-targeted gene and RNA-based therapeutic strategies for modulating neurodegeneration and neuroinflammation. Abbreviations: AAV (adeno-associated virus), GFAP (glial fibrillary acidic protein), Iba1 (ionized calcium-binding adapter molecule 1 (Iba1), MBP (myelin basic protein), IGF-1 (insulin-like growth factor 1), BDNF (brain-derived neurotrophic factor), siRNA (small interfering RNA), ASO (antisense oligonucleotide), ALS (amyotrophic lateral sclerosis), BBB (blood–brain barrier), CNS (central nervous system), SOD1 (superoxide dismutase 1), and C9orf72 (chromosome 9 open reading frame 72).

Therapeutic Strategy	Approach	Target Glial Cells	Key Effects/Applications	References
Viral vector-based gene delivery	AAV vectors with glial-specific promoters (GFAP, Iba1, and MBP)	Astrocytes Microglial cells Oligodendrocytes	Cell-specific gene expression with long-term, and low-immunogenic delivery	[305,306,307]
	AAV-mediated IGF-1 or BDNF overexpression	Astrocytes	Neuroprotection, reduced excitotoxicity, and synaptic support	[308,309]
	Engineered AAVs	Microglial cells	Shift toward anti-inflammatory and homeostatic phenotype	[310]
RNA-based therapies	siRNAs and ASOs	Astrocytes Microglial cells Neurons	Post-transcriptional gene silencing and transcript modulation	[312,313]
	ASOs targeting *Sod1* and *C9orf72*	Glial cells Neurons	Reduction in toxic proteins and RNA foci in ALS	[314,315]
	Clinically optimized ASOs/siRNAs	CNS	BBB penetration and suitability for repeated administration	[316,317]

**Table 4:** Glial cell transplantation strategies for neuroregeneration and restoration of CNS homeostasis. Abbreviations: iPSCs (induced pluripotent stem cells), OPCs (oligodendrocyte precursor cells), MLCs (microglia-like cells), ALSP (adult-onset leukoencephalopathy with axonal spheroids and pigmented glia), and GM2 (GM2 ganglioside).

Therapeutic Strategy	Approach	Target Glial Cells	Key Effects/Applications	References
Astrocyte transplantation	Astrocyte precursor cells iPSC-derived astrocytes	Astrocytes	Improved neuronal viability Reduced gliosis Axonal regeneration Potential for personalized autologous therapy	[318,319,320,321,322,323]
OPC transplantation	Oligodendrocyte precursor cells iPSC-derived OPCs	Oligodendrocytes	Migration to demyelinated regions Differentiation and remyelination Restoration of nerve conduction and functional improvement	[324,325,326,327,328]
Microglial transplantation	Hematopoietic stem cells iPSC-derived microglia MLCs	Microglial cells	Restoration of CNS homeostasis Myelin integrity Reduction in axonal spheroids Modulation of inflammation Potential therapy for ALSP and Sandhoff disease	[329,330,331,332,333,334]

**Table 5:** Nanotechnology- and targeted delivery-based strategies for glia-focused modulation of neuroinflammation and CNS therapy. Abbreviations: BBB (blood–brain barrier), PNP (polymeric nanoparticle), RVG (rabies virus glycoprotein), siRNA (small interfering RNA), miRNA (microRNA), EV (extracellular vesicle), MSC (mesenchymal stem cell), EXOmotif (exosomal motif), TLR4 (Toll-like receptor 4), TOP1 (topoisomerase 1), and MS (multiple sclerosis).

Therapeutic Strategy	Approach	Target Glial Cells	Key Effects/Applications	References
Nanoparticle-mediated delivery	PNPs Liposomes	Astrocytes Microglial cells	Cross BBB through receptor- mediated transcytosis Modulation of NF-κB, JAK/STAT, and MAPK pathways	[335,336,337,338,339,340,341,342,343,344,345]
Extracellular vesicles/Exosomes	MSC-derived or engineered exosomes	Astrocytes Microglial cells	Modulation of neuroinflammation Promotion of neuroprotection Rewire glial phenotypes	[346,347,348,349,350,351,352]
BrainShuttle™ and nanobody platforms	Engineered antibodies or nanobodies targeting BBB receptors	Microglial cells	Rgulation of pro-inflammatory pathways	[353,354,355]
Targeted TOP1 inhibition	Topoisomerase 1 inhibitors (topotecan and camptothecin) delivered through β-glucan-coated DNA origami (TopoGami)	Microglial cells Myeloid cells	Suppression of microglial/ macrophage inflammation Attenuation of neuroinflammation	[356]

## Data Availability

No new data were created or analyzed in this study.

## Abbreviations

The following abbreviations are used in this manuscript:

24p3R	24p3 receptor
A1	Astrocyte subtype 1
A2	Astrocyte subtype 2
AAV	Adeno-associated virus
AD	Alzheimer’s disease
AKT	Protein kinase B
ALS	Amyotrophic lateral sclerosis
ALSP	Adult-onset leukoencephalopathy with axonal spheroids and pigmented glia
AMPA	α-amino-3-hydroxy-5-methyl-4-isoxazolepropionic acid
ANLS	Astrocyte-neuron lactate shuttle
AP-1	Activator protein 1
APC	Antigen-presenting cell
ApoE	Apolipoprotein E
AQP4	Aquaporin 4
Arg1	Arginase 1
ARIA	Amyloid-related imaging abnormalities
ASC	Apoptosis-associated speck-like protein containing a CARD
ASD	Autism spectrum disorder
ASO	Antisense oligonucleotide
ATF4	Activating transcription factor 4
ATF6	Activating transcription factor 6
ATP	Adenosine triphosphate
Aβ	Amyloid beta
BBB	Blood–brain barrier
BDNF	Brain-derived neurotrophic factor
C1q	Complement component 1q
C3	Complement component 3
C3a	Complement component 3a
C3b	Complement component 3b
C5a	Complement component 5a
C9orf72	Chromosome 9 open reading frame 72
CARD	Caspase activation and recruitment domain
CCI	Chronic constriction injury
CCL2	CC chemokine ligand 2
CCL5	CC chemokine ligand 5
CD11b	Cluster of differentiation 11b
CD11c	Cluster of differentiation 11c
CD16	Cluster of differentiation 16
CD18	Cluster of differentiation 18
CD20	Cluster of differentiation 20
CD200	Cluster of differentiation 200
CD200R	Cluster of differentiation 200 receptor
CD206	Cluster of differentiation 206
CD25	Cluster of differentiation 25
CD32	Cluster of differentiation 32
CD36	Cluster of differentiation 36
CD4	Cluster of differentiation 4
CD40	Cluster of differentiation 40
CD44	Cluster of differentiation 44
CD52	Cluster of differentiation 52
CD55	Cluster of differentiation 55
CD59	Cluster of differentiation 59
CD8	Cluster of differentiation 8
CD80	Cluster of differentiation 80
CD86	Cluster of differentiation 86
CIPN	Chemotherapy-induced peripheral neuropathy
CK1	Casein kinase 1
CNS	Central nervous system
COX	Cyclooxygenase
CR3	Complement receptor 3
CR4	Complement receptor 4
CREB	cAMP response element-binding
CSF1	Colony-stimulating factor 1
CSF1R	Colony-stimulating factor 1 receptor
CX3CL1	Chemokine (C-X3-C motif) ligand 1
CX3CR1	Chemokine (C-X3-C motif) receptor 1
Cx43	Connexin 43
CXCL1	Chemokine (C-X-C motif) ligand 1
CXCL10	Chemokine (C-X-C motif) ligand 10
DAM	Disease-associated microglia
DAMP	Damage-associated molecular pattern
DAP12	DNAX activating protein of 12 kDa
DAPC	Dystrophin-associated protein complex
DDIT3	DNA damage-inducible transcript 3
DNA	Deoxyribonucleic acid
Dp71	Dystrophin protein of 71 kDa
EAAT1	Excitatory amino acid transporter 1
EAAT2	Excitatory amino acid transporter 2
EAE	Experimental autoimmune encephalomyelitis
eIF2α	Eukaryotic initiation factor 2 alpha
ER	Endoplasmic reticulum
ERAD	ER-associated degradation
ERK1/2	Extracellular signal-regulated kinases 1 and 2
EV	Extracellular vesicle
EXOmotif	Exosomal sorting motif
FasL	Fas ligand
Fizz1	Found in inflammatory zone 1
GABA	Gamma-aminobutyric acid
GDNF	Glial cell line-derived neurotrophic factor
GFAP	Glial fibrillary acidic protein
GJA1	Gap junction alpha 1
GLAST	Glutamate aspartate transporter
GLT-1	Glutamate transporter 1
GM2	GM2 ganglioside
GM-CSF	Granulocyte-macrophage colony-stimulating factor
HD	Huntington’s disease
HIF-1α	Hypoxia-inducible factor 1 alpha
HMGB1	High-mobility group box 1
Iba1	Ionized calcium binding adapter molecule 1
iC3b	Inactive complement component 3b
ICAM-1	Intercellular adhesion molecule 1
IFN-α/β	Interferon alpha/beta
IFN-γ	Interferon gamma
IFNGR	Interferon-γ receptor
IGF-1	Insulin-like growth factor 1
IgG	Immunoglobulin G
IgG1	Immunoglobulin G subtype 1
IKK	IκB kinase
IKKα	IκB kinase alpha
IKKβ	IκB kinase beta
IKKγ	IκB kinase gamma
IL-10	Interleukin 10
IL-12	Interleukin 12
IL-18	Interleukin 18
IL-1α	Interleukin 1 alpha
IL-1β	Interleukin 1 beta
IL-33	Interleukin 33
IL-34	Interleukin 34
IL-4	Interleukin 4
IL-6	Interleukin 6
IL-6R	Interleukin 6 receptor
iNOS	Inducible nitric oxide synthase
IRAK	Interleukin-1 receptor-associated kinase
IRE1α	Inositol-requiring enzyme 1 alpha
IRF3/7	Interferon regulatory factors 3 and 7
ITAM	Immunoreceptor tyrosine-based activation motif
IκBα	Inhibitor of κB alpha
JAK	Janus kinase
JAK/STAT	Janus kinase/signal transducer and activator of transcription
JNK	c-Jun N-terminal kinase
LCN2	Lipocalin 2
LINGO-1	Leucine-rich repeat and Ig domain-containing Nogo receptor-interacting protein 1
LPS	Lipopolysaccharide
LTP	Long-term potentiation
M1	Microglia subtype 1
M2	Microglia subtype 2
mAb	Monoclonal antibody
MAC	Membrane attack complex
MAG	Myelin-associated glycoprotein
MAI	Myelin-associated inhibitor
MAPK	Mitogen-activated protein kinase
MBP	Myelin basic protein
MCT1	Monocarboxylate transporter 1
MCT2	Monocarboxylate transporter 2
MDD	Major depressive disorder
MEGF10	Multiple EGF-like domains 10
MERTK	MER tyrosine kinase
MHC-I	Major histocompatibility complex class I
MHC-II	Major histocompatibility complex class II
miRNA	MicroRNA
MCAO	Middle cerebral artery occlusion
MLC	Microglia-like cell
MMP-2	Matrix metalloproteinase 2
MMP-9	Matrix metalloproteinase 9
MOG	Myelin oligodendrocyte glycoprotein
Mr BMT	Microglia replacement by bone marrow transplantation
MS	Multiple sclerosis
MSC	Mesenchymal stem cell
mTOR	Mechanistic target of rapamycin
mTORC1	Mechanistic target of rapamycin complex 1
MyD88	Myeloid differentiation primary response 88
NADPH	Nicotinamide adenine dinucleotide phosphate
NFAT	Nuclear factor of activated T-cells
NF-κB	Nuclear factor kappa-light-chain-enhancer of activated B cells
NgR1	Nogo receptor 1
NLRP3	NOD-like receptor family pyrin domain-containing 3
NMDA	N-methyl-D-aspartate
NMOSD	Neuromyelitis optica spectrum disorder
NO	Nitric oxide
Nogo-A	Neurite outgrowth inhibitor A
NOX2	NADPH oxidase 2
NSAID	Non-steroidal anti-inflammatory drug
OPC	Oligodendrocyte precursor cell
OXPHOS	Oxidative phosphorylation
P2X7	Purinergic receptor P2X, ligand-gated ion channel 7
P2Y12	Purinergic receptor P2Y12
P2Y6	Purinergic receptor P2Y6
p38	p38 protein
p75^NTR^	p75 neurotrophin receptor
PAMP	Pathogen-associated molecular pattern
PD	Parkinson’s disease
PERK	Protein kinase RNA-like ER kinase
PGE_2_	Prostaglandin E_2_
PI3K	Phosphoinositide 3-kinase
PKC	Protein kinase C
PLCγ	Phospholipase C gamma
PLP	Proteolipid protein
PLP1	Proteolipid protein 1
PML	Progressive multifocal leukoencephalopathy
PNP	Polymeric nanoparticle
PPARγ	Peroxisome proliferator-activated receptor gamma
PRR	Pattern recognition receptor
Rac1	Ras-related C3 botulinum toxin substrate 1
RAGE	Receptor for advanced glycation end products
RhoA	Ras homolog family member A
RNA	Ribonucleic acid
RNAi	RNA interference
RNS	Reactive nitrogen species
ROCK	Rho-associated coiled-coil-containing protein kinase
ROS	Reactive oxygen species
RVG	Rabies virus glycoprotein
S100B	S100 calcium-binding protein B
scRNA-seq	Single-cell RNA sequencing
Sca1^−^	Stem cell antigen-1 negative
siRNA	Small interfering RNA
SIRP1A	Signal regulatory protein alpha
SOCS3	Suppressor of cytokine signaling 3
SOD1	Superoxide dismutase 1
Src	Proto-oncogene tyrosine-protein kinase Src
STAT	Signal transducer and activator of transcription
STAT1	Signal transducer and activator of transcription 1
SUCNR1	Succinate receptor 1
Syk	Spleen tyrosine kinase
TAK1	TGF-β activated kinase 1
TBI	Traumatic brain injury
TDP-43	TAR DNA-binding protein 43
TGF-β	Transforming growth factor beta
TIR	Toll/interleukin-1 receptor
TLR	Toll-like receptor
TLR2	Toll-like receptor 2
TLR3	Toll-like receptor 3
TLR4	Toll-like receptor 4
TLR9	Toll-like receptor 9
TMEM119	Transmembrane protein 119
TNFR	Tumor necrosis factor receptor
TNFR1	Tumor necrosis factor receptor 1
TNF-α	Tumor necrosis factor alpha
TOP1	Topoisomerase 1
TRAF6	TNF receptor-associated factor 6
TRAIL	Tumor necrosis factor-related apoptosis-inducing ligand
TREM2	Triggering receptor expressed on myeloid cells 2
TRIF	TIR-domain-containing adapter-inducing interferon
TROY	Tumor necrosis factor receptor superfamily member 19
UPR	Unfolded protein response
VCAM-1	Vascular cell adhesion molecule 1
VEGF	Vascular endothelial growth factor
XBP1	X-box binding protein 1
Ym1	Chitinase-like protein 3
